# How I Follow Hodgkin Lymphoma in First Complete (Metabolic) Remission?

**DOI:** 10.3390/medicina60020344

**Published:** 2024-02-19

**Authors:** Vibor Milunović

**Affiliations:** Division of Hematology, Clinical Hospital Merkur, 10000 Zagreb, Croatia; vibor96@gmail.com

**Keywords:** Hodgkin lymphoma, cancer survivors, adverse effect, brentuximab vedotin, comorbidity

## Abstract

Hodgkin lymphoma is characterized by a high cure rate in the modern era of medicine regardless of stage, but patients suffer from a high risk of comorbidity associated with the administered therapy. The main aim of this review article is to assess and analyze the various comorbidities associated with Hodgkin lymphoma and address the survivorship of patients, including fertility, secondary cancers due to cardiovascular toxicity, and quality of life. Furthermore, this review explores the optimal strategy for detecting relapse. The treatment paradigm of Hodgkin lymphoma has shifted, with a paradigm shift toward achieving a high cure rate and low toxicity as a standard of care in this patient population. Checkpoint inhibitors, especially nivolumab, in combination with chemotherapy are increasingly being studied in the first line of therapy. However, their long-term toxicity remains to be assessed in longer follow-up. In conclusion, Hodgkin lymphoma survivors, regardless of their treatment, should be followed up individually by a multidisciplinary survivorship team in order to detect and properly treat the long-term side effects of therapy.

## 1. Introduction

Hodgkin lymphoma (HL) is a rare hematological malignancy, with the incidence estimated as 2.5 cases per 100,000 men and women [1]. The median age at diagnosis is 39 years old, with a high 5-year relative survival rate of 88.9%. HL is certainly a story of success, with the breakthrough of the MOPP (mechlorethamine, vincristine, procarbazine, and prednisone) regimen showing for the first time that cancer malignancy can be treated with chemotherapy and offering a cure for 50% of patients [2]. This regime was deemed by the American Society of Clinical Oncology (ASCO) as the first modern and important advance in oncology [3]. With the development of novel regimens, such as ABVD (doxorubicin, bleomycin, vinblastine, and dacarbazine) and BEACOPPesc (bleomycin, etoposide, doxorubicin, cyclophosphamide, vincristine, procarbazine, and prednisone), the outcomes of patients have improved greatly. However, a new paradigm in HL has arisen, which focuses on how to maintain a low mortality rate and reduce comorbidities in this patient population. The main aim of this review is to describe the long-term consequences of HL treatment and provide strategies for clinicians on how to deal with them.

## 2. The Short Overview of Hodgkin Lymphoma Treatment

To understand the follow-up of patients with HL, a brief overview of the ever-changing treatment paradigm of HL is presented.

While the treatment of early, favorable HL typically involves around two cycles of ABVD and INRT, there is a heated debate about the treatment of early, unfavorable (two cycles of ABVD plus two cycles of BEACOPPesc followed by 30 Gy of INRT) and advanced disease, which is guided by positron emission tomography–computed tomography (PET-CT) [4]. There is a heated debate regarding the approach for patients with advanced disease, specifically about the use of ABVD or BEACOPPesc in this clinical scenario. Skoetz et al. performed a meta-analysis on 3427 subjects included in five randomized controlled trials [5]. The patients treated in the BEACOPPesc arm had better progression-free survival (PFS) (HR = 0.54, 95% CI 0.45–0.64) and overall survival (OS) (HR = 0.74, 95% CI 0.57–0.97). However, this meta-analysis suffers from publication bias due to the time of publication and novel paradigms such as four cycles of BEACOPPesc, which have shown the best outcomes in advanced HL as demonstrated by the HD18 trial in PET-negative patients [6]. Nevertheless, there is still heated ongoing discussion about the use of this regimen. The choice of chemotherapy depends on the physician’s and patient’s preference, or the country. Central Europe tends to use BEACOPPesc as a standard of care, unlike Italy, Great Britain, and the US, which are more prone to ABVD. An Italian group conducted an RCT comparing four cycles of BEACOPPesc followed by four cycles of BEACOPPbaseline with ABVD in 331 patients with unfavorable characteristics [7]. The primary endpoint was freedom from first progression, favoring BEACOPPesc with a 7-year freedom from progression rate of 85% and 73%, respectively (*p* = 0.004). However, there was no difference in event-free survival (EFS). This lack of EFS may be attributed to high acute toxicity, treatment-related mortality (TRM), and secondary malignancies. In conclusion, despite the initial HL-related outcomes, the acute and long-term toxicities limit this protocol. However, further trials are needed to examine the current BEACOPPesc GHSG standard to ABVD.

The greatest breakthrough is the addition of brentuximab vedotin (BV), an antiCD30 antibody–drug conjugate, to the AVD backbone as demonstrated by the ECHELON-1 RCT comparing this new regimen to ABVD on 1134 subjects with advanced HL [8]. The 6-year PFS rate was 82.3% in the experimental group compared to 74.5% in the ABVD group (HR = 0.68, 95% CI 0.53–0.86). However, the significance of the ECHELON-1 study lies in the OS benefit, which had not been seen for decades in HL research. The 6-year OS rate was 93.9% in the BV group compared to 89.4% in the ABVD group (HR = 0.59, 96 CI% 0.40–0.88). Furthermore, this regimen is not PET driven, as it overcomes Deauville 4 interim PET in terms of PFS and OS. This regimen should become the standard of care in the treatment of advanced HL, although it has not been tested against BEACOPPesc.

Another attractive strategy is certainly PET-driven treatment [9,10,11,12,13,14]. While interim PET-negative patients benefit from such strategies as the omission of bleomycin, interim PET-positive patients face challenges in terms of PFS rate and the risk of death, representing the unmet need in the field of treating HL [9]. The PET guided trials are shown in Table 1, titled “PET Adapted Approach in Hodkin Lymphoma” [10].

## 3. How to Detect the Relapse of Hodgkin Lymphoma

It is estimated that around 15% of patients with early disease and 30% of patients with advanced disease develop relapse of HL and are treated by salvage therapy followed by autologous stem cell transplantation [15,16]. In a meta-analysis concerning advanced disease, Dalal et al. estimated 5-year PFS rates of 58–81% for ABVD and rates of 83–96% for BEACOPP variants [17]. Furthermore, even though treatments in RCTs are well defined, “real-life” treatments are heterogenous and their impact on HL relapse remains unexplored. The European Society of Medical Oncology (ESMO) recommends anamnesis, physical examination and a laboratory analysis during follow-up (grade IVB) [18]. It is important to note that follow-up is frequent after achieving complete remission (CR) or complete metabolic remission (CMR). The British guidelines are somewhat vague, stating that follow-up should be based on patient and physician preferences, but are against routine imaging [19]. In US National Comprehensive Cancer Care (NCCN) hospitals, computed tomography (CT) scans are performed every 6 months in the context of clinical trials [20]. However, it is important to note that other physicians, mainly from Europe, often follow these guidelines. The American College of Radiology (ACR) recommends MSCT surveillance every 6 months for 2 years and then once per year for 3 years [21]. A possible rationale for the NCCN and ACR guidelines is that HL relapse is most common in the first two years after first achieving CR, and functional cure of HL is defined as being in CR for 5 years. Earlier studies, such as the one by Zinzani et al., considered this notion outdated [22]. Gandigota et al. performed serial CT surveillance in early HL on 78 patients [23]. None of the patients relapsed, with nine false positive scans. As expected, this mode of surveillance was not cost-effective, totaling USD 296,817. The MD Anderson’s retrospective experience regarding early HL (*n* = 179) showed that only 0.2% of relapses were detected by routine surveillance, with 463 scans needed to detect one relapse [24]. Lee at al. estimated a positive predictive value (PPV) of 28.6 for CT scans in detecting relapses with a high economic burden (USD 61,820.48 to detect one relapse) [25]. With the advent of PET-CT and the Lugano criteria (despite not recommending routine imaging during follow-up), this imaging technique is increasingly being used in HL surveillance [26]. In a study with 67 patients who were routinely followed up via PET-CT, Mochikova et al. reported a true positive scan in 9% of cases, while it was inconclusive in 17.9% of cases [27]. It is important to note that the value of PET-CT increased, if clinically indicated, to 18.5% (RR = 0.21, *p* = 0.02). Another retrospective study included 211 patients who were routinely followed up, and 81 patients showed clinical indication for PET-CT [28]. The true positive rate of PET-CT was only 5%, rising to 13% if clinically indicated (*p* < 0.05). The largest study in this area was conducted by the GELTAMO group (*n* = 640) [29]. The patients were divided into five groups, who underwent clinical assessment (*n* = 202), CT every 3 months (*n* = 58), CT every 6 months (*n* = 232), PET-CT every 3 months (*n* = 43), or PET-CT every 6 months (*n* = 82). During follow-up, 68 patients relapsed. It is important to note that in the majority of cases, the suspicion was raised by the patient themselves, based on their symptoms (lymphadenopathy, constitutive symptoms) (61.8%), with the PPV being 64%, in contrast to CT (PPV = 59%) and PET-CT (PPV = 47%). This study included long-term follow-up, with the PFS and OS rates not being statistically significant among the groups, thus prompting the authors to conclude that a clinical approach toward this patient group is the method of choice. Furthermore, imaging techniques are a concern due to exposure to radiation. The median millisievert (mSV) at two years is highest for CT scans performed every 3 months (126.68) compared to routine clinical imaging (19.9). These data are worrisome due to the risk of secondary cancers because of unnecessary exposition to radiation, especially in younger and vulnerable groups of HL patients [30]. 

Due to the pitfalls of CT or PET-CT imaging, Picardi et al. designed a randomized control trial comparing ultrasound and chest X-ray imaging to routine PET-CT follow-up on 300 patients with advanced disease [31]. The study met its endpoint, showing no difference in the detection of relapses due to the techniques used. The PPV for ultrasound imaging was 91% compared to 73% for PET-CT, with the NPV being 99% and 100%, respectively. This is “a proof-of concept” study showing that easily accessible and cost-effective techniques with minimal irradiation could be used in everyday clinical work.

One other feature of monitoring HL is late relapses [32]. An analysis of Swedish registry data including 2242 HL patients found 58 late relapses, with the incidence being 2.7% at 10 years, 4% at 15 years, and 5.4% at 20 years. When compared to HL patients in CR, the outcomes were poor, with the 10-year OS rate being 51%, which is similar to early relapses. It is important to note that the treatment strategy in these cases is unknown. On the other hand, the incidence of late relapses has diminished since the year 2000, indicating the improvement in first-line treatment. The GHSG analyzed their trials and found 45 patients with late relapses [33]. The outcomes of these patients were better than those with early relapses (HR = 0.7, *p* = 0.03). Contrary to patients with early relapses, autologous stem cell transplantation was used in a minority of cases and patients mostly received BEACOPPesc or ABVD.

In conclusion, expert clinical assessment combined with patients’ symptomatology is the preferred method for detecting relapse, along with the biopsy of a suspicious lesion. If the biopsy is positive, CT or PET-CT should be used to restage the disease. However, there remains an unmet need in this area, concerning iPET-positive patients who are prone to relapses. There is no scientific consensus on how to follow up with these patients.

## 4. Survivorship among HL patients with First Complete Remission 

As shown above, the majority of patients will be cured from HL after first-line therapy. Yet, a question has been raised regarding whether these patients have excess morbidity and mortality. Núñez et al., in their retrospective single-institution study (*n* = 383), analyzed the standard mortality ratio (SMR) not related to HL (1967–2020) [34]. Female sex (SMR = 5.61) and age younger than 30 years at diagnosis (SMR = 12.5) were risk factors for death. Interestingly, despite the progress in the treatment of HL, the SMR in the years 2000–2022 was 3.88 (95% CI = 2.41–6.24), which was greater than before the year 2000 (SMR = 2.73), indicating that the treatment of HL has a great impact on the OS of these patients. This is a cautionary tale suggesting that hematologists do not adequately follow up with this vulnerable group of patients. 

## 5. Secondary Solid Tumors

### 5.1. Secondary Breast Cancer

Ibrahim et al. performed a meta-analysis of 34 studies on secondary breast tumor [35]. The median age at HL diagnosis was 23.7 years, while the median age at secondary breast cancer (SBC) diagnosis was 35 years. The latency between HL diagnosis and SBC diagnosis was 17.7 years. The rate of excess cases was 22.9 per 10,000 persons per year, with a relative risk of 8.23. As expected, the risk was highest in young HL survivors, i.e., children, while older patients (21–30-year-old) had a relative risk of 5.3 (95% CI = 1.9–16.6). It is important to note that women older than 40 (at HL diagnosis) were not at risk for SBC. Concerning treatment modalities, radiotherapy was the main risk factor for the development of this secondary tumor (RR = 4.7). Interestingly, combined treatment with an alkylating agent was also a risk factor for SBC (RR = 5.65). However, there are limitations to this meta-analysis due to the treatment modalities being explored (mantle cell radiotherapy) and the inclusion of studies of historical importance. With the advent of novel radiotherapeutic strategies such as EFRT and INFRT, the risk has become lower (OR = 3.25), while the SBC occurrence in patients receiving a smaller field of radiotherapy is relatively low (20-year incidence rate = 3.5%) [36]. It is important to note that other radiation variables, such as the total number of grays delivered or the fractions of radiotherapy, play a role in SBC occurrence. Concerning the outcome, SBC confers poorer overall survival when compared to sporadic breast cancer regardless of disease stage [37].

The primary solution to this problem is prevention and screening [38]. Routine mammograms are recommended despite additional radiation. Concerning breast magnetic resonance imaging, the evidence is based on expert opinions, though no advantage has been shown for this patient population. However, there is another issue that needs to be assessed, i.e., patients’ adherence to screening. 

In a study by Diller et al., 40% of patients were not aware of the increased risk for SBC, with only 47% having received a mammogram in the last two years [39]. This indicates the need for proper education by multidisciplinary teams and evidence-based programs to offer appropriate care to female HL survivors.

### 5.2. Secondary Pulmonary Cancer

Lorigan at al. performed a systematic review on secondary pulmonary cancer in HL survivors (*n* = 32,951) [40]. In general, RR was 2.9 with an absolute excess risk of 9.7. Both mediastinal radiation greater than 5 Gy (RR = 7.2) and use of alkylating agents (RR = 4.3) were predictors of this neoplasm with an additive effect (RR = 7.2 95% CI = 2.8–21.6%). However, the magnitude of pulmonary cancer was much more pronounced in cigarette smokers (at least one pack per day). In this population, irradiation carried an RR of 20.2, with the RR of alkylating agent chemotherapy being 16.8. The additive effect was greatest with an RR of 49.1 (95% CI = 15.1–187). The latency period was between 5 and 9 years, with the risk persisting for up to 20 years. Concerning the outcomes, an analysis using the SEER database compared secondary lung cancer to primary lung cancer [41]. Non-small cell lung cancer (NSCLC) was diagnosed in 466 HL survivors, while small cell lung cancer (SCLC) was diagnosed in 93 cases. The median latency of diagnosis was associated with the stage, with local pulmonary cancer having a median latency of 7 years. Furthermore, the year of diagnosis was significant concerning the stages at diagnosis, with patients being diagnosed with an early stage from 2000 to 2016, indicating that chemotherapy, different radiation techniques, and survivorship programs may have changed the approach to HL survival. However, HL survivors with NSCLC had a worse prognosis than the comparison group irrespective of stage, while there was no difference in survivors with SLCL.

As shown above, secondary lung cancer is an important secondary solitary tumor in HL survivors that confers poor prognosis. There are several prevention techniques to avoid this dismal prognosis. The first primary prevention is offering patients multidisciplinary support to stop smoking. Another possible secondary prevention is using low-dose CT to detect lung cancer in the early stage. A meta-analysis by Hoffman et al. included 96,559 subjects screened by means of MSCT or chest X-ray [42]. Lung cancer screening detected more stage I cases (RR = 2.93) and resulted in reduced mortality (RR = 0.84), with 265 cases needed to be screened to avoid one lung cancer death. The false positive rate was 8%. Yet, these findings cannot be translated directly into HL survival for various reasons; a feasibility study of low-dose CT screening to detect lung cancer in this population is being carried out on 200 survivors (NCT04396119). If positive, it could change our paradigm in detecting this fatal complication.

## 6. Secondary Hematological Malignancies

Two of the most common secondary hematological malignancies following chemotherapy are therapy-related acute myeloid leukemia (tAML) and therapy-related myelodysplastic syndrome (tMDS), which confer dismal prognosis [43]. In three prospective trials comparing the MOPP and ABVD regimens, the 15-year actuarial risk for developing AML in the ABVD group was 0.7% compared to 2.4% in the MOPP group [44]. It is important to note that irradiation in the ABVD group did not have an additive effect on the development of secondary hematological malignancies. A Stanford group evaluated retrospectively three clinical trials (*n* = 754), with the rate of t-AML being 3.2% [45]. However, the difference was significant among the trials, with none of the patients developing t-AML in the Stanford V group. The authors attributed these findings to the cumulative dose of cytostatic drugs used in the various regimens, mainly mechlorethamine, melphalan, and procarbazine. The GHSG evaluated the rates of t-AML and t-MDS in 11,952 subjects in 11 RCTs [46]. The total rate of t-AML/MDS was 0.9% (*n* = 106). Unlike secondary solid tumors, the latency to secondary hematological malignancies was shorter, occurring during the first three years after CR. In the multivariate analysis, age, number of BEACOPPesc (≥4), and extended-field radiotherapy were independent risk factors. The median OS was poor at 7.2 months. However, younger patients could be treated with allogeneic stem cell transplantation, with the median OS not yet determined.

Furthermore, HL survivors are prone to develop secondary non-Hodgkin lymphoma, as demonstrated by a Cochrane meta-analysis (frequency of 0.9%) [47]. In conclusion, secondary hematologic malignancies are rare events in HL survivorship, unlike secondary solid cancers, but they represent a clinical challenge due to the lack of primary or secondary prevention and limited therapeutic strategies.

## 7. Cardiovascular Toxicity

One of the largest studies on late cardiovascular toxicity in HL survivors was conducted by a Dutch collaborative group involving 2524 HL survivors, with a median follow-up of 20.3 years [48]. It is important to note that this is a historical cohort study due modalities such as mantle cell radiotherapy being used in the treatment. A total of 1713 events were recorded, including chronic heart disease (CHD) in 401 patients, valvular heart disease (VHD) in 374 patients, and heart failure (HF) in 140 patients. When graded according to the Common Terminology Criteria for Adverse Events, most events were grade 3 or higher. When compared to the general population, the standardized incidence ratio (SIR) was higher, with the SIRs for CHD and HF being 3.2 and 6.8, respectively. HL survivors younger than 25 at the age of diagnosis had statistically significant elevated SIRs for cardiovascular toxicity than older survivors. The risks for CHD and HD were higher during the 20-to-47-month follow-up after the first CR, indicating the latency of these events. In the multivariate analysis, mediastinal radiotherapy, intensity of radiation, anthracycline use, and smoking status at diagnosis were significant prognostic factors for cardiovascular events.

Another study on cardiovascular disorders was carried out by the EORTC and LYSA groups using the self-reported measure, Life Situation Questionnaire (LSQ), in subjects enrolled in RCTs [49]. A total of 4735 patients were eligible for the LSQ (all baseline data regarding treatment were obtained), with 1919 responders. The majority of patients were treated in the years between 1995 and 2004 with a combined modality of treatment. Ischemic heart disease was reported in 24% of cases, followed by congestive heart failure in 21% of cases and VHD in 14% of cases. Another adverse event was arrythmia, occurring in 17% of cases. As reported by the study using Dutch registry data, these were late onset events, with a gradual increase of up to 25 years after the initial treatment. In the multivariate analysis, the mean dose of radiation to the heart and the mean dose of anthracyclines were important factors. Furthermore, increases in these variables were associated linearly with cardiovascular events. Hodgson et al. performed a large population study involving HL survivors, with the primary endpoint being hospitalization due to a cardiac event (*n* = 3.964, median age at HL diagnosis = 35) [50]. Doxorubicin-containing chemotherapy, followed by mediastinal irradiation, was the most prominent risk factor (HR = 1.8). Furthermore, the authors analyzed patients receiving only ABVD. The risk for cardiac-related hospitalization at 10 years was 5.5% for males and 3.3% for females, showing that the most used regimen in HL has serious cardiac morbidity. A small retrospective study compared acute and early cardiac toxicities between the ABVD and BEACOPP regimens by measuring heart indices using ultrasound before and one year after therapy [51] Although the BEACOPP regimen was associated with the detection of cardiac function indices, the result was not clinically significant. Due to the relative novelty of this regimen, longer follow-up with a large cohort of patients is needed to assess the long-term cardiac morbidity associated with it. In this clinical scenario, prevention has a role. One prevention strategy involves the correction of additional risk factors (smoking, obesity, diet, and modification of risk factors such as blood pressure). Furthermore, in patients suffering from HL with underlying cardiac disease or risk factors, treatment with liposomal doxorubicin can be the treatment of choice in the so-called PBVD regimen [52]. Liu et al. analyzed this regimen in 46 HL patients, with 32 of the patients having a history of cardiovascular disease or risk factors. The overall response rate was 91%, with 25 CRs in this group. Similar results were obtained in the group of patients without underlying cardiovascular disorders or risk factors. The 3-year PFS rate for the entire group was 70% with an OS rate of 82%, showing no difference between the groups. It is important to note that long-term outcomes were similar to those seen with ABVD. Cardiac events were recorded in nine patients, but they were all grade I or II. This study serves as a “proof of concept” demonstrating the feasibility of liposomal doxorubicin. However, this strategy should be evaluated in a larger number of patients in future research. 

However, it appears that routine cardiac follow-up is mandatory in HL survivors. As mentioned above, HL survivors are at risk for coronary artery disease, but are usually asymptomatic. Coronarography is not the procedure of choice since its risks outweigh its benefits. Unfortunately, traditional non-invasive testing methods such as stress echo or radionuclide testing are inappropriate due to their low sensitivity. Novel techniques such as CT and MR angiography are promising, but the coronary artery calcium score may be the diagnostic tool of choice in this setting due to its high sensitivity and specificity [53]. It is recommended to use this tool every 5 years starting at 10 years after the first CR. Screening for VHD depends on age, and it is recommended that HL survivors older than 45 years are screened at the time of first CR, while in younger patients, screening should start at 10 years after the first CR. Yet, the impact of screening is controversial because there is no evidence-based approach to screening HL survivors with VHD. A multidisciplinary team is needed to weigh the benefits and risks of valvular replacement on an individual basis. Concerning arrythmia, routine ECG is warranted at each follow-up visit because it can easily detect heart conduction disorders.

In conclusion, cardiovascular toxicity is one of the major reasons underlying the morbidity and mortality of HL survivors, and an experienced cardiologist must be involved as part of the follow-up of these patients.

## 8. Sexual Gonadal Toxicity

Due to the fact that HL survivors are young, fertility is of great importance in regard to survivorship. As expected, male fertility and female fertility are managed differently due to physiological and pathophysiological differences; thus, this section is divided into two subsections [54].

### 8.1. Male Survivors

One of the particularities of the male gonadal system is the quality of sperms prior to therapy. An early report comes from the GHSG trials including 158 patients [55]. Various abnormalities such as oligo-, astheno-, and azoospermia were found in 70% of cases, causing the authors to conclude that infertility was present prior to treatment. Interestingly, advanced disease was an independent predictor of these findings, indicating that HL itself may affect the fertility of male HL patients due to an unknown mechanism regulation. However, a larger study by the EORTC group involving 474 patients with early HL did not confirm these findings [56]. The authors divided the patients into three different groups according to their sperm quality based on the number and motility of sperms. The majority of patients had intermediate sperm (49%) or good (41%) sperm quality. Once again, the independent factors for poor sperm quality were related to HL characteristics. Due to the abundant literature on sperm quality in HL patients prior to therapy, a meta-analytical approach is needed to concisely answer this question. However, the main impact on male fertility is the choice of first-line chemotherapy. Amin et al. performed a meta-analysis of five studies (*n* = 1344) to compare the gonadal toxicity of the ABVD and BEACOPP regimens [57]. At 6 months following ABVD, the majority of patients had changes in sperm quality (oligospermia in 38% of cases and azoospermia in 40% of cases). These findings were associated with the number of drug cycles given. Yet, this was a transient finding since at 24 months following ABVD, the majority of patients recovered their sperm quality. Following treatment with the BEACOPP variants, 89% of patients became azoospermic and 11% of cases showed dyspermia. A negligible number of HL survivors recovered spermatogenesis. The authors pointed out that the main limitation was that neither of these studies assessed fertility preservation. Although cryopreservation is a gold standard prior to gonadotoxic therapy, the real-life situation is different, as reported by the EORTC group [58,59]. By using a specific self-reported questionnaire, 913 HL survivors were identified. Surprisingly, cryopreservation was only carried out in 40% of patients (*n* = 363). Concerning pregnancies, 334 HL survivors wanted a child, with spontaneous success in 206 cases. Among HL survivors who had undergone the procedure, artificial reproductive techniques (ART) resulted in 48 successful pregnancies. The main limitation of this study was that it covered the years 1974–2004, thus not representing real-world data on HL treatment in the present time. Every patient with HL and a wish for a child, regardless of the planned treatment type, must be offered cryopreservation.

### 8.2. Female Survivors

The main issue among HL female survivors is premature ovarian failure (POF) diagnosed in younger patients with secondary amenorrhea or unsuccessful pregnancies. The main etiological factor is the type of chemotherapy given. Machet et al. investigated the association of various protocols with fertility in 67 patients, with two controls per case [60]. The main outcome was the number of pregnancies and births. In the ABVD group (*n* = 37), a total of 32 pregnancies were recorded, with 26 births; there was no significant difference compared to the controls, thus corresponding to 81% of patients with at least one birth. No association between ABVD and pregnancy termination was found. These data support that the ABVD regimen is not gonadotoxic and fertility is preserved. The GHSGG group studied 762 women participating in the HD13-HD15 RCTs [61]. The probability of amenorrhea at 4 years after therapy was highest in patients with advanced HL who were treated with BEACOPP in the HD15 trial and was correlated with age (25% risk at the age of 25 vs. 50% risk at the age of 30). Severe menopausal symptoms were present in women older than 30 years of age, with a fivefold increase in risk for POF. Yet, only 48.9% of the patients took hormone replacement therapy. Concerning pregnancies and births, 51.9% of patients expressed a desire for a child. Only 15% of the HD15 subjects reported pregnancy after 4 years of follow-up. These results establish the BEACOPP variants as a truly gonadotoxic therapy in advanced HL. One of the possible solutions to this problem is to offer ABVD therapy or a PET-adapted approach, as shown in the AHL2011 RCT [62]. A second solution is to provide a referral to a specialized gynecologist for fertility preservation methods, such as in vitro fertilization for embryo cryopreservation, mature oocyte cryopreservation, or cryopreservation of ovarian tissue [63]. However, these are time-consuming techniques that can lead to a delay of therapy, and a multidisciplinary team must weigh the risks and benefits for individual patients. Concerning the use of gonadotropin-releasing hormone analogs (GnRH-a) among HL patients receiving BEACOPP therapy, the GHSG phase II trial was stopped after the interim analysis due to not detecting any differences between the treatment arms receiving estradiol, follicle-stimulating hormone and anti-Mullerian hormone, translating into the possibility of the ovarian follicle preservation rate being 0% [64].

## 9. Brentuximab Vedotin-Induced Neuropathy

Brentuximab vedotin-induced neuropathy (BVIN) has emerged as a novel toxicity after the introduction of BV + AVD as a first-line therapy for advanced Hodgkin lymphoma due to antibody–-conjugate mechanism of action, i.e., off-target effects of monomethyl auristatin E [65]. In a pivotal trial, it occurred in 67% of subjects with grades 2 and 3, with a rate of 20% and 11%, respectively [8]. Complete resolution occurred in 43% of patients, with the one-grade improvement rate being estimated to be 24% during the last follow-up. It is important to note that BV was discontinued in 10% of cases. However, the real-world data show a higher incidence of this AE [66]. In a study by Steiner et al. involving 179 patients receiving BV + AVD, the rate of BVIN was 75%, with the grade 3 rate being 12%. One of the possible risk factors for grade 3 BVIN was a higher age at the time of treatment, irrespective of the cumulative BV dose. Improvement in BVIN occurred in 34% of patients, with complete resolution achieved in 35% of cases. Concerning the management of BVIN, the most common approach is a reduction in BV dose or its omission (starting a new type of chemotherapy). However, the main cause of worry is whether a reduction in BV dose compromises the outcomes of patients. The decrease in cumulative BV dose was not associated with the rate of CMR, but it was statistically, marginally associated with an inferior PFS. Concerning patient expectations regarding BVIN (*n* = 381), a recent survey involving patients with different treatment profiles showed that the outcomes in terms of PFS and OS were more important than this AE, indicating that BVIN is acceptable in this patient population [67]. However, data on quality of life and BVIN are lacking, so the full impact and burden of BVIN is not known.

## 10. Fatigue and Quality of Life

HL survivors suffer from decreased functioning, mainly fatigue, as demonstrated by the GHSG in their early trials when compared to controls [68]. A recent survey involving 120 HL survivors showed severe levels of general fatigue (28%), physical fatigue (26%), and reduced activity (22%) when measured using the Multidimensional Fatigue Inventory [69], In contrast, 21 patients reported no fatigue-related symptoms. Concerning associations with treatment type, patients who received ABVD and radiotherapy reported the highest levels of fatigue compared to patients treated with BEACOPP. Further findings of this study included a decline in quality of life (Qol), with 16% of patients reporting very poor QoL and 36% of patients reporting low physical function. In a Norwegian study involving 298 patients (treated between 1997 and 2016), 42% of patients reported chronic fatigue that was associated with a lower QoL [70]. A systematic review on fatigue in HL survivors included 22 studies, with most of them being cross-sectional studies [71]. The estimated rates of fatigue were 26–30% when compared to 10% in the general population. It is important to note that these two prospective studies showed decreased fatigue during follow-up. Concerning treatment, the majority of studies found an association with the modality of treatment. In the analysis of fatigue and comorbidities, patients with cardiac or respiratory dysfunction showed greater levels of fatigue. In conclusion, fatigue in HL survivors is an important comorbid issue and, although the exact mechanism is not known, it is likely to be multifactorial, ranging from cytokine dysregulation to comorbidities such as depression and anxiety [72].

Another important aspect of survivorship is anxiety and depression. Unfortunately, no data exist for HL survivors, but a recent study examined this issue in lymphoma survivors (*n* = 224, *n* of HL patients = 102) [73]. The rates of anxiety and depression among all patients included in the study were 17% and 12.3%, respectively. Furthermore, 8% presented with concomitant anxiety and depression. Higher anxiety levels were more prevalent in HL survivors (*p* = 0.037). Although the rate was seemingly low, a comorbid psychiatry illness affected all SF36 QoL variables (*p* = 0.01). Concerning intervention, psychological support did not have an impact on these comorbidities, unlike prescribed psychiatric drugs (*p* = 0.001). In conclusion, HL survivors should be screened for anxiety and depression using simple questionnaires, such as the Hospital Depression and Anxiety Scale, in order to intervene early when patients are presented with these comorbid psychiatric conditions [74].

## 11. Thyorid Dysfunction

Hodgkin lymphoma survivors are at high risk for thyroid malfunction [75]. A retrospective single-institution study involving 237 female HL survivors found hypothyroidism in 30% of cases and thyroid nodules in 6.8% of cases. Chemotherapy was not a risk factor, but neck radiotherapy was (RR = 5.0). It is recommended that endourologists are part of the team caring for this patient population, with regular screening for thyroid hormone status and, occasionally, thyroid gland ultrasound.

## 12. Where Are We Now in 2023? Maximizing Cure Rate and Minimizing Toxicity

### 12.1. Advanced Disease

#### 12.1.1. BrECADD

To minimize the toxicities of BEACOPPesc for the treatment of advanced HL, the GHSG developed two new regimens, BrECAPP (brentuximab vedotin, etoposide, doxorubicin, procarbazine, and prednisone) and BrECADD (brentuximab vedotin, etoposide, doxorubicin, cyclophosphamide, dacarabazine, and dexamethasone) and tested them in a phase II trial involving 104 subjects with advanced HL [76]. The primary endpoints were complete response and complete remission. The complete response rates were 86% in the BrEACAPP group and 88% in the BrECADD group. Due to the fact that the null hypothesis was rejected, complete remission was excluded as a primary co-endpoint. Concerning PFS, the 18-month rates were 95% and 89%, respectively, for the BrEACAPP and BrECADD groups. Toxicity was mainly hematological, with few infections. The peripheral neuropathy incidence was low, mostly associated with stages I and II, unlike the result for the BV + AVD regimen. Due to the fact that BrECADD had a more adequate safety profile, especially concerning organ toxicity, this regimen was preferred by the GHGG to be explored in a phase III trial. The non-inferiority HD21 trial was conducted with 1482 subjects being randomized into the BEACOPPesc arm or BrECADD arm in a 1:1 ratio [77]. It was a PET-adapted trial with the number of cycles of each regimen being varied based on the iPET status. This trial defined the co-primary endpoints as non-inferiority to BEACOPPesc and treatment-related morbidity (TRMB). TRMB was defined as acute hematological toxicity and acute organ toxicity. Per protocol, irradiation was allowed in EOT PET-positive patients with a lymph node size ≥ 2.5 cm. The three-year PFS rate was 92.3% for the BEACOPPesc group compared to 94.9% for the BrECADD group, with the HR for the inferiority margin being 1.02. After a median follow-up of 40 months, the median OS was not reached in either of the groups. Concerning TRMB, acute hematological toxicities and infections were more profound in the BEACOPPesc group (*p* < 0.0001), while TRMB was lower in the experimental group (c-RR = 0.72, 95%CI 0.65–0.79, *p* < 0.0001). Concerning the AEs of interest, sensory peripheral neuropathy was more pronounced in the BECOPPesc arm, with gonadal function estimated based on follicle-stimulating hormones being within the normal range in the BrECADD arm in both male and female patients. This regimen is characterized by appropriate efficacy and low TRMB and is now considered the standard of care by the GHSG for advanced HL. An updated analysis of the HD21 trial showed CMR rates of 80% and 82%, respectively, at the end of treatment [78]. Concerning interim PET, 5% of patients achieved CRM and were treated with four cycles of therapy. In the BrECADD group, the 3-year PFS rate was 94.9% compared to 92.3% in the group treated with BEACOPesc (HR = 0.63, 95% CI = 0.42–0.94). When stratified by interim PET, in PET-negative patients, the 3-year PFS rate was 97.1% and 93.6%, respectively. In PET-positive patients, the 3-year PFS rate was 93.5% in the experimental group compared to 90.6% in the standard treatment group. In the sub-analysis, the HRs regarding patients’ characteristics favored BrECADD, except for older age. Concerning TRMB, the RR was 0.72 and in favor of the experimental group. The authors concluded that the BrECADD regimen resulted in unprecedented, advanced HL disease control in the RCTs, further establishing it as a standard of care in the real-world setting. To examine TRMB, an analysis of the pregnancy rate was performed at the end of treatment [79]. The majority of male patients had cryopreservation (76.4%) when compared to females (46.1%). A total of 97 patients with at least one pregnancy was reported, with the percentage being higher in the experimental group. It is important to note that cryopreservation was used in a minority of patients, showing lower gonadal toxicity with an increase in female pregnancy rate. However, due to the short follow-up, the long-term toxicities of this regimen are unknown.

Yet, among hematologists treating HL, the BrECADD regimen remains a topic of debate. The main reason for this is its acute toxicities. A total of 24% of subjects received at least one red cell transfusion, while 17% needed at least one platelet transfusion [77]. Another concern is the lack of reporting on other serious side effects, such as infections or febrile neutropenia. Furthermore, since the HD21 study has not been published as a full article, the complete toxicity profile of the regimen remains unknown. It is expected that BrEACADD will become the standard of care for advanced HL in Central Europe, whereas other hematologists may opt for different regimens.

#### 12.1.2. Nivolumab

One of the major axes involving Reed–Stenberg cells is the PD-1/PD-L axis, which is encoded by 9p24.1 chromosome amplification and responsible for immune evasion and T-cell exhaustion [80]. Nivolumab, an anti-PD-1 monoclonal antibody, targets this axis, thereby restoring the immune microenvironment and leading to HL cell death. Checkpoint inhibitors are attractive options in first-line treatment of HL, and several preliminary studies have incorporated nivolumab as a possible therapeutic option. The first study by Ramchandren et al. was CheckMate 205, a Cohort D phase II trial exploring nivolumab in patients with newly diagnosed advanced HL (*n* = 51) [81]. All patients received four doses of nivolumab, followed by 12 cycles of nivolumab-AVD. The primary endpoint was safety. Apart from hematological toxicities and febrile neutropenia (10%), immune-related AEs were of interest. Nivolumab showed an adequate safety profile, with a minority of patients exhibiting immune AEs such as ALT and AST elevations and hepatitis. The most common AE was dysfunction of the thyroid gland, which occurred in 13 cases. Concerning efficacy, the CMR rate was 80% at the end of treatment. However, the follow-up was short, being only 12.1 months, and the modified 9-month PFS rate was 92%. This was a “proof-of-concept” study of nivolumab efficacy in first-line treatment of HL, leading to the phase III SWOG S1286 trial comparing nivolumab + AVD to BV-AVD in 994 patients, with the primary endpoint being PFS [82]. It is important to note that pediatric patients aged 12 or older were included in the trial (24%). At the first interim analysis after a median follow-up of 12.1 months, the 1-year PFS rate in the nivolumab group was 94% compared to 86% in the BV group (HR = 0.48, 95% CI = 0.27–0.87, *p* = 0.0005). Furthermore, less than 1% of patients needed consolidative radiotherapy. Concerning Aes, hematological toxicity was predominant, with a similar occurrence of febrile neutropenia across the groups (5.6% vs. 6.4%). Immune-related AEs of interest were rare. The nivolumab group had more thyroid gland-related AEs (10%), while, as expected, the BV group had PN. However, there are some limitations to this RCT. The first limitation is certainly its short follow-up, and currently, it is impossible to assess whether patients in the nivolumab group will maintain response. Even though the authors claimed that one of the study aims is to harmonize pediatric and adult HL protocols, currently, BV-APE-PC remains the standard of care in pediatric patients with advanced HL [83]. 

#### 12.1.3. The Role and Improvement of Radiotherapy in HL

Historically, mantle and inverted Y radiotherapies were the standard of care in the treatment of HL, but due to late toxicities, these modalities were abandoned [84]. Furthermore, the need for these modalities to treat advanced HL diminished with the rise of novel regimens. The main question remains if they can be omitted in early HL. The long-term follow-up of the HD16 RCT showed inferior outcomes among patients treated with ABVD only [85]. In the PET-negative group, combined modality resulted in a superior 5-year PFS rate of 94.2% compared to 86.7% in the ABVD group (HR = 2.05, inferiority margin = 3.01). The incidence of previous HL localization relapse was 2% in the combined modality group compared to 10.4% in the ABVD group (*p* = 0.0005 The analysis of the SEER database by Koshy et al. on 12,247 patients with stage I or II HL showed a steady decline in the use of radiotherapy, with only 43.7% of patients receiving it in the era 2004–2006, which affected the outcome [86]. Irradiated patients had significantly better OS, with a 5-year OS rate of 87% compared to only 76% in patients who did not receive radiotherapy. The authors did not find an elevated risk for secondary malignancy in irradiated subjects. However, one of the major RCTs is certainly the HD17 trial, which explored the effect of radiotherapy after a 2 × 2 regimen [87]. In this study, 548 patients were randomized to combined modality, while 552 patients were irradiated according to end-of-treatment PET-CT. There was no difference in 5-year progression-free survival, it being 97.3% and 95.1%, respectively, with the inferiority margin being 2.2%, suggesting that early, unfavorable HL should not be treated with combined therapy. These striking differences in findings could be explained by various biases such as different therapies used, not stratifying patients into risk groups, and other biases inherent to SEER data. However, given the increasing importance of real-world data in science, the findings by Koshy et al. should not be simply disregarded, especially in stage I HL patients. Regarding bulky disease, some hematologists are inclined to refer their patients to radiotherapists to consolidate the disease, but it remains questionable whether this is evidence-based. Gallamini et al. in the GITIL/FIL HD 0607 Trial performed a second randomization according to disease bulk and radiotherapy [13]. There was no significant improvement in the 3-year PFS rate, it being 93% and 97%, respectively. Zinzani et al. found a statically insignificant increase in PFS in irradiated patients with bulky disease in the HD0801 Study [12]. In conclusion, irradiation of bulky disease can be safely omitted, improving safety by avoiding unnecessary lung injury. Yet, the largest study concerning PET-guided therapy was the HD15 trial, despite the primary objective being the number of BEACOPPesc cycles as a standard of care in advanced HL [88]. The radiation therapy of 30 Gy was limited to PET-positive areas equal to or greater than 2.5 cm and Deauville scores of 1 and 2. The mains bias of this study is the inclusion of patients with a Deauville score 3, despite research indicating CMR [89]. A total number of 175 patients were irradiated. The rates of 2-year PFS were 86.2% and 92.5%, with only 11% of patients having primary refractory disease or early relapse, respectively. This indicates adequate disease control in this population, becoming the standard of care in ongoing clinical trials and clinical practice. Subsequent research, particularly the primary HD18 study, established a Deauville score of 3 as CMR, resulting in fewer patients being irradiated [6]. Based on this research, radiotherapy in advanced HL lymphoma is in decline, offering reduced toxicity and appropriate disease control. Another option may include the extirpation of PET-positive lymph nodes to differentiate false-positive PET residual mass and limit further therapy in HL patients in CMR. Regarding ABVD, in the RATHL study, only 20 patients were irradiated at the researcher’s discretion, making it impossible to comment on its impact on the outcome [9]. Gallamini et al. performed a randomized clinical trial on radiotherapy in three groups, stratified based on pretreatment bulky disease (*n* = 296) [89]. Following CMR in ABVD, the authors randomized the subjects to a radiation therapy group with no further treatment. The 6-year PFS was not significantly different among the groups (*p* = 0.44), with 6-year PFS rates of 93% and 91%, respectively. In a multivariate analysis for radiotherapy, no significant factor was identified. All these data show a real decline in radiotherapy, except in specific clinical scenarios, with minimization of toxicity in HL.

## 13. Conclusions

**Figure 1:**
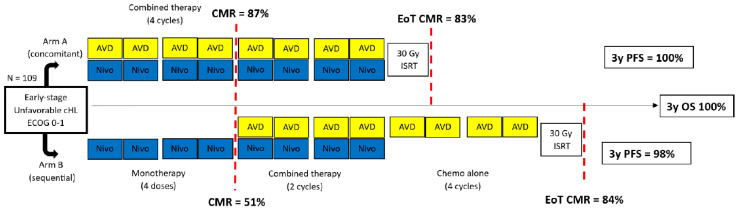
The Study Design and Results from the NIVAHL Study (adapted from Vassilakopoulos et al. [90]. Creative Common rights respected).

Despite the robust data, research in HL (phase II studies) treatment is changing with the incorporation of BV in early Hodgkin lymphoma, and checkpoint inhibitors in first-line treatment [90]. One of the largest studies on BV in early-stage, unfavorable HL was the BREACH study (*n* = 170) [91]. The initial treatment consisted of two cycles of either ABVD or BV-AVD, followed by interim PET. Subsequent therapy included two more cycles, but it was not PET-adapted. The primary outcome was the rate of interim PET negativity in the BV group, which was achieved with 82.3% in the BV group compared to 75.4% in the ABVD group. There was no difference in iPET-negative groups among the regimes, unlike in iPET positive groups, where the 2-year PFS was 93.8% and 71.8%, respectively. This suggests that a BV-based regimen may overcome this negative prognostic factor. Regarding checkpoint inhibitors, most data are available on nivolumab. The Phase II NIVAHL study, which included 109 patients, is illustrated in Figure 1 [92].

To the best of the author’s knowledge, the NIVAHL study is the largest phase II randomized trial exploring nivolumab in early stage, unfavorable HL with 109 patients. It is important to note that treatment solely with nivolumab, as shown in arm B, yielded lower CMR rates than nivolumab combined with AVD (51% vs. 87%). This indicates that using this agent alone does not control the disease properly and requires a chemotherapy backbone. The long-term results are impressive, with a 3-year PFS and OS rate of 100% in arm A, which is unprecedented in HL, indicating a paradigm shift in treating early-stage, unfavorable HL. Concerning immune-related adverse events, the regimen was feasible, with hypothyroidism being the main event, occurring in 21% of patients. Upper respiratory tract disorders were common but self-limited. 

The follow-up of HL patients after first achieving CR or CMR should be continuous and life-long due to the possibility of relapse and long-term AEs. In the follow-up process, the inclusion of the treating hematologist is not sufficient and a multidisciplinary survivorship team consisting of other specialists, such as a cardiologist, should be established. As outlined above, the treatment modalities of patients vary over the years and the follow-up plan should be tailored on an individual basis (the type of chemotherapy regimen, the type of radiation, and the expected long-term AEs). Despite the high cure rate of HL, early- and late-onset morbidities should be the central point during the follow-up of this vulnerable patient population. The paradigm of treatment for HL is changing, with a focus on maximizing the cure rate and minimizing toxicity, possibly leading us to a new era in our approach to HL.

## Figures and Tables

**Table 1:** Interim PET Guided Randomised Clinical Trials in Advanced Hodgkin Lymphoma.

Trial	Regimen	n	PFS (%)	OS (%)
GHSG HD 18 trial [6]	BEACOPPescx2 if iPET+ randomization to BEACOPPesc x4–6	217	89.7%(5y)	96.4% (5y)
	BECOPPescx2 if PET positive BEACOPPesc+R4–6	217	88.1% (5y)	93.9% (5y)
	BEACOPPescx2 if PET-BEACOPPesc2	501	92.2% (5y)	97.7% (5y)
	BEACOPPescx2 if PET-BEACOPP escx4–6	504	90.8% (5y)	95.4% (5y)
LYSA AHL 2011 [11]	BEACOPPesc2 if iPET-ABVDx4	319	85.7% (5y)	96.4% (5y)
	BEACOPPesc2 if iPET-BEACOPPesc4	49	NA	NA
	BEACOPPesc6 regardless of iPET	401	86.2% (5y)	95.2% (5y)
RATHL [9]	ABVDx2 if iPET-AVDx4	470	84.4 (3y)	97.2% (3y)
	ABVD if -PET-ABVDx4	465	85.7 (3y)	97.6% (3y)
	ABVDx2 if iPET+ BEACOPPesc21/14 (number of cycles varied)	172	67.6%(3y)	87.8%(3y)
Southwest Oncology Group S0816 [14]	ABVDx2 if iPET negative ABVDx4	271	82% (2y)	NA
	ABVDx2 if iPET+ BEACOPPescx6	60	64% (2y)	NA

## Abbreviations

HL	Hodgkin lymphoma
MOPP	mechlorethamine, vincristine, procarbazine, and prednisone
ASCO	American Society of Clinical Oncology
ABVD	doxorubicin, bleomycin, vinblastine, and dacarbazine
BEACOPPesc	bleomycin, etoposide, doxorubicin, cyclophosphamide, vincristine, procarbazine, and prednisone
INRT	involved-node radiotherapy
PET-CT	positron emission tomography–computed tomography
PFS	progression-free survival
OS	overall survival
HD	Hodgkin disease
EFS	event-free survival
GHSG	German Hodgkin Study Group
BV	brentuximab vedotin
AVD	doxorubicin, vinblastine, and dacarbazine
RCT	randomized clinical trial
PET	positron emission tomography
ESMO	European Society of Medical Oncology
US	United States
NCCN	National Comprehensive Cancer Care
CT	computed tomography
ACR	American College of Radiology
MSCT	multislice computed tomography
USD	Unites States dollar
PPV	positive predictive value
GELTAMO	group of lymphomas/autologous bone marrow transplantation
mSV	millisievert
NPV	negative predictive value
iPET	interim positron emission tomography
SMR	standard mortality ratio
CI	confidential interval
SBC	secondary breast cancer
RR	relative risk
Gy	gray
SEER	surveillance, epidemiology, and end results
NSLC	non-small cell lung cancer
SCLC	small cell lung cancer
tAML	therapy-related acute myeloid leukemia
tMDS	therapy-related myelodysplastic syndrome
CHD	chronic heart disease
VHD	valvular heart disease
HF	heart failure
CR	complete remission
EORTC	European Organisation for. Research and Treatment of Cancer
LYSA	The Lymphoma Study Association
LSQ	Life Situation Questionnaire
PBVD	lyposomal doxorubicin, bleomycin, vinblastine, and dacarbazine
MR	magnetic resonance
ART	artificial reproductive techniques
POF	premature ovarian failure
GNRH-a	gonadotropin-releasing hormone analogs
BVIN	brentuximab vedotin-induced neuropathy
CMR	complete metabolic remission
AE	adverse event
QoL	quality of life
SF36	36-Item Short Form Health
BrECAPP	brentuximab vedotin, etoposide, doxorubicin, procarbazine, and prednisone
BrECADD	brentuximab vedotin, etoposide, doxorubicin, cyclophosphamide, dacarbazine, and dexamethasone
TRMB	treatment-related morbidity
HR	hazard ratio
PD-1/PD-L1	programmed death-1, programmed death-ligand 1
ALT	alanine transaminase
AST	aspartate transferase
BV-APE-PC	brentuximab vedotin, doxorubicin, bleomycin, vincristine, etoposide, prednisone, and cyclophosphamide
